# Objective Sleep Characteristics and Factors Associated With Sleep Duration and Waking During Pediatric Hospitalization

**DOI:** 10.1001/jamanetworkopen.2021.3924

**Published:** 2021-04-01

**Authors:** Robyn Stremler, Samantha Micsinszki, Sherri Adams, Christopher Parshuram, Eleanor Pullenayegum, Shelly K. Weiss

**Affiliations:** 1 Lawrence S. Bloomberg Faculty of Nursing, University of Toronto, Toronto, Ontario, Canada; 2 The Hospital for Sick Children (SickKids), Toronto, Ontario, Canada

## Abstract

**Importance:**

Children’s sleep may be affected by hospitalization, yet few objective determinations of sleep patterns are reported for children in intensive care or general medicine units. There is limited research on relationships between sleep in hospital and child (eg, age, pain), treatment (eg, medications, nurse presence), or environmental (eg, noise, light, type of unit) factors.

**Objective:**

To determine sleep quantity and patterns in hospitalized children and determine factors associated with sleep quantity and nighttime waking for children in hospital.

**Design, Setting, and Participants:**

This was a prospective cross-sectional study of children admitted to a general pediatric unit or a pediatric intensive care unit at a pediatric quaternary teaching hospital in Toronto, Ontario, Canada, from October 2007 to July 2008. Participants included children aged 1 to 18 years who were expected to stay in hospital for at least 2 nights. Demographic data, information about the hospital stay and illness, and usual sleep habits were collected. Children wore an actigraph for 1 to 3 consecutive days and nights and completed a sleep diary. Sound and light meters were placed at the bedside. Data analyses took place in April 2009.

**Main Outcomes and Measures:**

The primary outcome was the mean number of minutes of child nighttime sleep from 7:30 pm to 7:29 am. Sleep variables were averaged over days and nights recorded (mean [SD] days and nights of wear, 2.54 [0.71]) and examined for associations with sleep quantity and patterns, as well as hazard of waking in the night.

**Results:**

Of 124 eligible children approached for inclusion, 69 children consented (35 [51%] female; 20 [29%] aged 1-3 years, 10 [14%] aged 4-7 years, 17 [24%] aged 8-12 years, and 22 [32%] aged 13-18 years; 58 [84%] in the general pediatric unit). Children aged 1 to 3, 4 to 7, 8 to 12, and 13 to 18 years obtained a mean (SD) of 444 (132), 475 (86), 436 (114), and 384 (83) minutes of nighttime sleep, respectively; mean (SD) number of night awakenings was 14 (3), 18 (3), 14 (8), and 12 (6), respectively. Children on general pediatric units slept 258 minutes more per night than children sleeping in the pediatric intensive care unit (95% CI, 165.16-350.56 minutes; *P* < .001), children admitted for planned surgery slept 123 minutes more than children admitted for exacerbations of chronic illness (95% CI, 49.23-196.01 minutes; *P* < .01), and children sleeping in rooms with other patients slept 141 minutes fewer than children in private rooms (95% CI, −253.51 to −28.35 minutes; *P* = .01). Sound events greater than 80 dB were significantly associated with increased risk of instantaneous waking (hazard ratio [HR], 1.35; 95% CI, 1.02-1.80; *P* = .04), as were light events greater than 150 lux (HR, 1.17; 95% CI, 1.01-1.36; *P* = .03), receiving a medication that promoted sleep (HR, 1.04; 95% CI, 1.00-1.08; *P* = .03), and having a nurse in the room for most or all of the night (HR, 1.08; 95% CI, 1.03-1.13; *P* = .003). Sleeping on the general pediatrics unit was significantly associated with decreased risk of instantaneous waking (HR, 0.81; 95% CI, 0.77-0.85; *P* < .001), as was being admitted for planned surgery (HR, 0.95; 95% CI, 0.91-0.99; *P* = .04), receiving a medication that promoted wakefulness (HR, 0.96; 95% CI, 0.93-0.995; *P* = .02), and sharing a room with another patient (HR, 0.78; 95% CI, 0.72-0.84; *P* < .001).

**Conclusions and Relevance:**

In this cross-sectional study of hospitalized children, children experienced considerable nighttime waking and sleep restriction to levels below national clinical recommendations at a time when they most needed the benefits of sleep. Given light and noise were the greatest contributors to nighttime waking in hospital, clinicians, administrators and hospital design experts should work together for solutions.

## Key Points

**Question:**

What is the quantity and patterns of sleep in hospitalized children, and what factors are associated with sleep duration and awakenings?

**Findings:**

In this cross-sectional study of 69 hospitalized children, those aged 1 to 3, 4 to 7, 8 to 12, and 13 to 18 years obtained only 444, 475, 436, and 384 minutes of nighttime sleep in hospital, respectively, which was less than at home and recommended sleep durations. Sound events exceeding 80 dB and light events greater than 150 lux were associated with increased hazards of nighttime waking.

**Meaning:**

During hospitalization, children may experience considerable sleep restriction and frequent waking from noise and light.

## Teaser

This cross-sectional study explores the quantity and patterns of sleep in hospitalized children to determine factors associated with sleep quantity and nighttime waking.

## Introduction

Sleep during pediatric hospitalization may be altered at a time when its benefits are needed most. Restorative sleep of adequate quantity is vital for physical health, with frequent awakenings and reduced sleep duration negatively affecting a child’s immune response,^1^ perception of pain,^2^ glucose regulation,^3^ and neuroendocrine function.^4^ Healthy children who experience sleep restriction are more likely to exhibit impaired cognitive function,^5,6,7^ inattentive behavior, reduced alertness, and decreased academic performance.^6,8,9^

Despite evidence that sleep is essential to overall health and healing, few studies have objectively examined sleep in hospitalized children,^10^ and available studies rely on subjective reports of child sleep quantity and quality by parent or other proxy observer.^11,12,13,14,15^ Studies objectively assessing pediatric sleep during hospitalization used polysomnography, electroencephalography, and actigraphy but are limited by small sample sizes and enrollment of specialized populations with cancer,^16,17,18,19^ burn injuries,^20,21,22,23^ neuromuscular blockade,^24^ and mechanical ventilation.^25^

During hospitalization, sleep may be affected by environmental factors (eg, light, noise), caretaking activities (eg, vital signs, parental presence),^14,15,26,27^ or the child’s physiological or behavioral responses (eg, pain, prehospitalization sleep habits).^12^ Frequent interruptions from staff or other environmental noise render complete cycling through sleep stages difficult for the hospitalized child.^10,11,12^ In studies in which patients were observed sleeping and those in which patients or parents reported the source of interruptions, sleep was frequently interrupted by nursing staff^14^ and alarms.^28^

Reports of noise and light levels on hospital units and pediatric intensive care units (PICUs) are consistently above recommended levels (<46 dB) and disturb sleep in adult patients.^29,30,31,32,33,34,35^ In PICU settings, baseline noise levels average 50 to 65 dB and are noisier during day vs night shifts.^27,36^ Exposure to bright light at nighttime has effects on body temperature, alertness, and circadian rhythm via alteration of melatonin production.^37,38^

Characterizing sleep patterns and identifying factors that affect sleep during hospitalization is an important step toward the development of strategies and interventions to decrease sleep disturbance and its negative health outcomes. The purpose of this study was to describe sleep quantity and sleep patterns in hospitalized children and determine variables associated with sleep duration and nighttime awakenings in hospitalized children.

## Methods

### Study Design

A prospective, cross-sectional study design was used. The Strengthening the Reporting of Observational Studies in Epidemiology (STROBE) reporting guidelines were used to generate the report of this study.^39^ Institutional research ethics board approval was received from the Hospital for Sick Children (SickKids). Adolescent patients provided their own informed consent, and informed consent was provided by the parents of younger hospitalized children.

### Setting and Sample

Children aged 1 to 18 years who were admitted to a general pediatric unit or PICU at a pediatric quaternary teaching hospital in Toronto, Ontario, Canada, were approached by a research assistant to participate if they were expected to stay in hospital for at least 2 nights. Patient rooms were predominantly single rooms with children only occasionally in a shared observation room. Children were excluded if they had a diagnosed sleep disorder, had limited or abnormal movements of both upper and lower extremities, if the consenting child or parent was unable to read or understand English, or if they were expected to die during the hospital admission.

### Measures

#### Sleep Variables

The primary outcome was the mean number of minutes of child nighttime sleep averaged over nights of recording (7:30 pm to 7:29 am). Sleep was measured using actigraphy, an objective, portable method for recording sleep data, and conducted by use of an actigraph. The actigraph detects and records continuous motion data with a wristwatch-sized microprocessor that senses motion with a piezoelectric linear accelerometer. Congruence between polysomnography and actigraphy indicates adequate validity and reliability when sleep is assessed in toddlers, older children, and adolescents,^40,41^ with excellent agreement. The Standards of Practice Committee of the American Academy of Sleep Medicine recommends at least three 24-hour periods of actigraph recording time.^42^ A sleep diary developed by an investigator (R.S.) was used to determine reasons for periods of little (eg, lying still) or complete (eg, removal of the actigraph) inactivity. Sleep diary data were used in interpretation of the actigraphy data. Secondary sleep outcomes included minutes of daytime (7:30 am to 7:29 pm) sleep, minutes awake after sleep onset, number of night wakings, length of longest sleep period during night, and length of longest sleep period during day.

#### Factors Associated With Sleep Duration and Awakenings

Factors examined for association with sleep outcomes included child factors (eg, age, reason for admission, pain score, usual sleep habits), treatment factors (eg, medications that increase wake time, induce sleep, or treat pain; nurse in the child’s room for most or all of the night [eg, child in a constant observation room]) and environmental factors (eg, parental presence in the room, unit type [eg, PICU, general pediatrics], room type [eg, single, shared], light, sound). Demographic information (eg, age, race) was collected from the child or parent at baseline, and information about the child’s health and treatment (eg, reason for admission, previous number of hospitalizations, medications known to affect sleep) was extracted from the medical record.

Children’s usual sleep habits were evaluated at enrollment using the Children’s Sleep Habits Questionnaire (CSHQ), a 45-item parent-reported sleep questionnaire developed to screen for the most common sleep problems in children, with total scores greater than 41 used to identify children with sleep disturbances.^43^ A sleep diary was used for the child or parent to record characteristics of the sleep environment for each night, including the type of unit, type of room (single or shared), and presence of nurse and parent. An age-appropriate pain score, scaled on a 0-10 metric, was included in the sleep diary to measure pain each morning. For children aged 1 to 6 years, the FLACC (Faces, Legs, Activity, Cry, Consolability) scale was completed by a research assistant,^44^ while children aged 7 to 12 years completed the Faces Pain Scale–revised^45^ and adolescents aged 13 to 18 years completed a visual analog scale to self-report their current pain intensity.^46^

Light and sound were measured using a MicroMini Sound and MicroMini Light Sensor (Ambulatory Monitoring Inc), respectively, which were time synchronized with the child’s actigraph and mounted on a portable plastic box kept near the bed through the study period. The sensors sampled and stored sound (40-85 dB) and light (0-4000 lux) levels, recording a value every minute based on the average of 8 readings per minute. These readings allow for examination of the number of minutes that light and sound exceeded World Health Organization recommended levels (>46 dB for noise and >150 lux for light levels), as well as the occurrence of light and sound events above recommended levels in the 2 minutes before, or the minute of, an awakening.^35,47^ Light and noise data were examined across nights from 7:30 pm to 7:29 am, as well as 10 pm to 6 am, to avoid the expected noise and light of shift change and to assess a time when most children, regardless of age, should be asleep.

### Procedure

Consenting children (adolescents, if able) or their parent completed a baseline questionnaire. Children wore MiniMotion Logger actigraphs (Ambulatory Monitoring Inc) on either the wrist or ankle continuously for 3 days and nights, reflecting usual length of stay and minimizing study burden. If the child was unable to continue for 3 days and nights (eg, discharged early), their actigraphy data remained in the analysis. Each morning, the parent or adolescent was visited by a research assistant who prompted them to complete the sleep diary, including morning pain scores and characteristics of the sleep environment.^48^ If children were transferred to other units during their participation in the study, they were followed to those units.

### Statistical Analysis

Actigraphy data were interpreted with autoscoring programs (Action4 software [Ambulatory Monitoring Inc]). Data were double entered into an Access 2007 (Microsoft Corporation) database and analyzed in SAS, version 9.1 (SAS Institute), to obtain the mean minutes of nighttime sleep, total sleep in 24 hours, number of nighttime awakenings, and longest stretch of nighttime sleep. To describe baseline characteristics and data related to sleep quantity and sleep patterns, frequencies (%) were used for categorical variables and means (SD) or medians (interquartile range [IQR]) for continuous variables. Finally, data from the light and sound meters were analyzed to obtain the mean minutes of light greater than 150 lux, minutes of sound greater than 46 dB, and minutes of sound greater than 80 dB. A generalized estimating equation was used to evaluate associations between minutes of nighttime sleep with child, treatment, and environmental variables, accounting for clustering within child to account for repeated measures across nights. A Cox proportional hazards model was used to evaluate hazards of nocturnal waking. Two-sided tests with a level of significance of *P* = .05 were used.

### Factors Associated With Sleep Outcomes

Minutes of nighttime sleep were recorded from 7:30 pm to 7:29 am to examine all minutes of sleep achieved by the child across the night. The following covariates were included in the generalized estimating equation: type of admission (ie, chronic, acute, or planned surgery), age of child (in years), CSHQ total score, unit type, parent presence in the room, nurse presence in the room, and single or shared room. Subsequently, medications related to wake, sleep, and pain, and total pain score were added. Finally, mean minutes of light greater than 150 lux, mean minutes of sound greater than 46 dB, and mean minutes of sound greater than 80 dB were also included.

Nocturnal waking was recorded from 10 pm to 6 am to best examine wakes across a period when participants of all ages should be asleep and when environmental influences on waking should be limited. Minute-by-minute data on sound, light, and wakefulness were analyzed using a Cox proportional hazards model with recurrent events. The event of interest was waking up, and only periods for which the child was asleep contributed to the analysis. The expected hazard of waking was analyzed using proportional hazards regression, where time was indexed as the time since the child last fell asleep. Covariates included in the regression were type of admission (ie, chronic, acute, or planned surgery), age of child (in years), unit type, CSHQ total score, parent presence in the room, nurse presence in the room, single or shared room, medications that promote wake (eg, steroids), medications for sleep (eg, chloral hydrate), medications for pain (eg, opioids), total pain score, time since the child first fell asleep for the night, whether there was sound between 46 and 80 dB or if there was sound greater than 80 dB in the current or previous 2 minutes, and finally, if there was light measured greater than 150 lux in the current or previous 2 minutes. Variance estimates accounted for clustering within the child.

## Results

Between October 2007 and July 2008, 124 eligible children and their parents were approached, and 69 (56%) children and their parents consented to inclusion. The majority of participants were White (n = 36 [54%]) or Asian (n = 18 [27%]), almost half male (n = 34 [49%]), distributed across age categories, and recruited from the general pediatric ward (n = 58 [84%]) or the PICU (n = 11 [16%]) (Table 1). Sleep data were available for 63 (91%) children who had been hospitalized for acute illness or trauma (n = 32 [47%]), chronic illness (n = 33 [48%]), or planned surgery (n = 3 [4%]). Of the 63 participants with sleep data, 42 (67%) had 3 nights of data, 13 (20%) had 2 nights of data, and 8 (13%) had 1 night of data (mean [SD], 2.54 [0.71] nights of sleep data; median [IQR], 3 [2-3] nights of sleep data). Participants missed nights of data collection due to early discharge from hospital (n = 19 [30%]), actigraphy malfunction (n = 2 [3%]), and study withdrawal (n = 1 [2%]). Mean scores in all age groups met the criteria on the CSHQ for previously existing problematic sleep (scores >41).^43^

**Table 1.** Baseline Characteristics of Hospitalized Children Aged 1 to 18 Years

Characteristic	No. (%)
Total No.	69
Sex	
Male	34 (49)
Female	35 (51)
Age, y	
1-3	20 (29)
4-7	10 (14)
8-12	17 (24)
13-18	22 (32)
Location of enrollment	
PICU	11 (16)
General pediatric unit	58 (84)
Type of admission	
Chronic illness	33 (48)
Acute illness or trauma	32 (47)
Planned surgery	3 (4)
Missing data	1 (1)
Race/ethnicity	
White	36 (54)
Asian	18 (27)
Black	5 (7)
Multiracial	4 (6)
Aboriginal	2 (2)
Hispanic	2 (2)
Parent’s age, mean (SD), y	
Male (n = 68)	39.2 (7.2)
Female (n = 65)	41.5 (7.5)
Age-appropriate grade (±1 grade)	
Yes	64 (93)
No	5 (7)
Prior hospitalizations in past 12 mo, mean (SD)	1.1 (1.5)
CSHQ Score >41^a^	60 (87)

Abbreviations: CSHQ, Children’s Sleep Habits Questionnaire; PICU, pediatric intensive care unit.

^a^ Scores greater than 41 indicate a problem with sleep.

### Sleep Outcomes

As children enrolled in the PICU were similar in age, sex, and type of admission to other hospitalized patients, and because of the 11 children recruited from the PICU, only 3 spent more than 1 night in the PICU before transfer to a lower acuity unit, mean sleep data were not disaggregated by unit type (Table 2). Children aged 1 to 3, 4 to 7, 8 to 12, and 13 to 18 years obtained a mean (SD) of 444 (132), 475 (86), 436 (114), and 384 (83) minutes of sleep, respectively, with mean (SD) number of awakenings at night ranging from 12 (5.5) to 18 (3.4) and the mean (SD) longest stretch of sleep ranging from 130 (48) to 133 (71) minutes. Sleep duration in all age categories was below both sleep amounts reported at home and national clinical recommendations for adequate sleep quantity,^49^ with the largest reductions in sleep quantity observed among the youngest age groups.

**Table 2.** Sleep Outcomes Among Hospitalized Children Aged 1 to 18 Years by Age Category

Sleep parameter	Age category			
	1-3 y	4-7 y	8-12 y	13-18 y
No. of patients	19	10	15	19
Nocturnal sleep time, mean (SD), min^a^	444 (132)	475 (86)	436 (114)	384 (83)
Parent-estimated usual sleep time prehospitalization, mean (SD), min	647 (77)	656 (54)	562 (73)	505 (71)
Deficit between mean usual and mean in-hospital sleep time, min	−203	−181	−126	−121
NSF recommended lower limit of nighttime sleep, min	660	600	540	480
Deficit between NSF recommended and mean in-hospital sleep time, min	−216	−125	−104	−96
24-h sleep over night 1 and day 2, mean (SD), min	599 (210)	445 (153)	514 (181)	445 (147)
No. reported	14	9	13	18
Length of longest nocturnal sleep, mean (SD), min^b^	130 (48)	131 (68)	133 (71)	131 (84)
No. reported	12	8	13	13
No. of night wakings, mean (SD)^a^	14 (3.3)	18 (3.4)	14 (7.5)	12 (5.5)
No. reported	12	8	13	13
Daytime sleep time, mean (SD), min^b^	216 (126)	74 (87)	69 (41)	82 (76)
No. reported	15	9	13	18
Length of longest daytime sleep, mean (SD), min^c^	65 (22)	30 (26)	33 (15)	26 (21)
No. reported	8	6	12	11
Average nocturnal sleep onset time	8:36 pm	9:08 pm	9:27 pm	10:21 pm

Abbreviation: NSF, National Sleep Foundation.

^a^ Nocturnal sleep time and night waking are defined as 7:30 pm to 7:29 am.

^b^ Daytime sleep time is defined as 7:30 am to 7:29 pm.

### Light and Sound

When light and sound were examined across the entire night (7:30 pm to 7:29 am), mean (SD) minutes of light greater than 150 lux ranged from 44 (32) to 100 (104) minutes (Table 3). Across the whole night, mean (SD) minutes of sound greater than 46 dB ranged from 84 (55) to 115 (77) minutes, while mean (SD) minutes of sound greater than 80 dB ranged from 32 (28) to 47 (40) minutes. During the time on the unit when noise and light were expected to be lowest (10 pm to 6 am), mean (SD) minutes of light greater than 150 lux ranged from 4 (9) to 35 (54) minutes, with higher levels noted in the adolescent group. During this time, mean (SD) minutes of sound greater than 46 dB and greater than 80 dB ranged from 27 (21) to 43 (35) minutes and 9 (8) to 15 (19) minutes, respectively.

**Table 3.** Minutes of Light and Sound Above Critical Levels at the Bedside of Hospitalized Children Aged 1 to 18 Years by Age Category

Light and sound	Age category, mean (SD)			
	1-3 y	4-7 y	8-12 y	13-18 y
No. of patients	16	10	15	19
Light >150 lux, min				
7:30 pm to 7:29 am	44 (32)	39 (60)	62 (66)	100 (104)
10 pm to 6 am	10 (20)	4 (9)	9 (17)	35 (54)
Sound >46 dB, min				
7:30 pm to 7:29 am	112 (59)	84 (55)	115 (77)	98 (70)
10 pm to 6 am	42 (28)	27 (21)	40 (35)	43 (35)
Sound >80 dB, min				
7:30 pm to 7:29 am	47 (40)	32 (28)	44 (41)	37 (45)
10 pm to 6 am	14 (13)	9 (8)	12 (15)	15 (19)

### Factors Associated with Sleep Outcomes

#### Minutes of Nighttime Sleep

Compared with children admitted for exacerbations of chronic illness, children admitted for planned surgery achieved 123 minutes more sleep each night (95% CI, 49.23-196.01 minutes; *P* < .01), even after adjusting for the other variables (Table 4). Children sleeping in shared rooms achieved 141 minutes less sleep than children sleeping in single rooms (95% CI, −253.51 to −28.35 minutes; *P* = .01). Children sleeping on general pediatric units slept 258 minutes more per night than children sleeping in the PICU (95% CI, 165.16-350.56 minutes; *P* < .01). No statistically significant associations were found between any of the other variables and amount of nighttime sleep.

**Table 4.** GEE Model of Nighttime Sleep Regressed Onto Child, Treatment, and Environmental Variables, and Hazard Model of Variables Associated with Nighttime Waking^a^

Variable	GEE model			Hazard model	
	Estimate (SE)	(95% CI)	*P* value	HR (95% CI)	*P* value
Intercept	147.04 (113.70)	(−75.81 to 369.88)	.20	NA	NA
Admission type					
Acute illness/trauma	53.28 (30.92)	(−7.33 to 113.90)	.08	1.00 (0.97 to 1.04)	.82
Planned surgery	122.62 (37.44)	(49.23 to 196.01)	<.01	0.95 (0.91 to 0.99)	.04
Chronic illness	1 [Reference]	NA	NA	1 [Reference]	NA
Child’s age, y	−4.12 (2.91)	(−9.82 to 1.59)	.16	1.00 (0.996 to 1.00)	.96
CSHQ total score	−1.22 (2.41)	(−5.94 to 3.49)	.61	0.99 (0.96 to 1.01)^b^	.30
Nurse presence in room					
Yes	−44.01 (23.92)	(−90.88 to 2.86)	.07	1.08 (1.03 to 1.13)	.003
No	1 [Reference]	NA	NA	1 [Reference]	NA
Parent presence in room					
Yes	9.02 (31.77)	(−53.24 to 71.29)	.78	0.98 (0.95 to 1.02)	.35
No	1 [Reference]	NA	NA	1 [Reference]	NA
Room type					
Shared	−140.93 (57.44)	(−253.51 to −28.35)	.01	0.78 (0.72 to 0.84)	<.001
Single	1 [Reference]	NA	NA	1 [Reference]	NA
Unit type					
General pediatrics	257.86 (47.30)	(165.16 to 350.56)	<.01	0.81 (0.77 to 0.85)	<.001
PICU	1 [Reference]	NA	NA	1 [Reference]	NA
Medication					
Promotes wakefulness	−27.98 (28.87)	(−84.56 to 28.60)	.33	0.96 (0.93 to 0.995)	.02
Promotes sleep	64.46 (46.91)	(−27.48 to 156.41)	.17	1.04 (1.00 to 1.08)	.03
Treats pain	−31.97 (52.28)	(−134.43 to 70.50)	.54	0.96 (0.93 to 1.00)	.05
Pain score^c^	−3.60 (3.55)	(−10.55 to 3.36)	.31	1.01 (0.999 to 1.01)	.09
Light event >150 lux	−0.19 (0.18)	(−0.55 to 0.17)	.30	1.17 (1.01 to 1.36)	.03
Sound event, dB					
>46	0.01 (0.36)	(−0.70 to 0.71)	.98	1.05 (0.87 to 1.27)	.60
>80	−0.15 (0.27)	(−0.68 to 0.38)	.58	1.35 (1.02 to 1.80)	.04

Abbreviations: CSHQ, Children’s Sleep Habits Questionnaire; GEE, generalized estimating equation; HR, hazard ratio; NA, not applicable; PICU, pediatric intensive care unit.

^a^ Nighttime sleep is defined as occurring 7:30 pm to 7:29 am, and nighttime waking as occurring 10 pm to 6 am.

^b^ Children’s Sleep Habits Questionnaire, in increments of 10.

^c^ In 2 minutes before or in the minute of an awakening.

#### Nighttime Waking

Table 4 also shows the multivariate hazard ratios (HRs), which estimate the instantaneous hazard of waking once the child was asleep between 10 pm to 6 am. Sound events greater than 80 dB were associated with a 35% increase in the hazard of waking (HR, 1.35; 95% CI, 1.02-1.80; *P* = .04), while light events greater than 150 lux were associated with a 17% increase in the hazard of waking (HR, 1.17; 95% CI, 1.01-1.36; *P* = .03). Receiving a medication that promoted wakefulness was associated with a 4% decrease in the hazard of waking (HR, 0.96; 95% CI, 0.93-0.995; *P* = .02), while receiving a medication that promoted sleep was associated with a 4% increase in the hazard of waking (HR, 1.04; 95% CI, 1.00-1.08; *P* = .03), likely reflecting the child’s state necessitating the medication. Sleeping in a room shared with another patient was associated with a 22% decrease in the hazard of waking (HR, 0.78; 95% CI, 0.72-0.84; *P* < .001), while having a nurse in the room for most or all of the night was associated with an 8% increase in the hazard of waking (HR, 1.08; 95% CI, 1.03-1.13; *P* = .003). Sleeping on the general pediatrics unit was associated with a 19% decrease in the hazard of waking (HR, 0.81; 95% CI, 0.77-0.85; *P* < .01). Being admitted for planned surgery was associated with a 5% decrease in the hazard of waking (HR, 0.95; 95% CI, 0.91-0.99; *P* = .04).

## Discussion

In this study, children admitted to general pediatrics and critical care units experienced a large sleep deficit, on average at least 2 hours fewer per night than both parent-reported levels at home before hospitalization and levels recommended by national sleep organizations. Health care professionals caring for children should be concerned with this amount of sleep deprivation given that acute effects of such sleep loss include impairment of physiological processes key to recovery, as well as low mood and decrements in cognitive processing, thereby affecting the child’s ability to cope with the psychological challenges of hospitalization.^33^ Although parents slightly overestimate their child’s sleep at home owing to unobserved wake time at night,^50^ the levels of sleep loss recorded remain concerning, particularly because actigraphy may overestimate sleep duration when there is low level of movement (eg, when lying in bed awake).^51^ Furthermore, amounts of sleep achieved during the day in this sample do not fully make up for lost nighttime sleep.

The present sample achieved a similar amount of nighttime sleep as in the study by Bevan et al^52^ with a smaller, similarly aged sample on a general pediatrics unit, although they found no differences in amount of sleep achieved based on whether the space was shared or for a single child. In the center for this study, shared rooms were those in which multiple patients were under constant observation and likely required more assessment and intervention from health care professionals.

Sleep quality was poor in hospital, as evidenced by the present sample’s frequent awakenings and limited ability to cycle through all stages of sleep. Although excess levels of light and noise have long been known to exist in hospital environments, to our knowledge, this study is the first to model the contribution of a comprehensive set of child, environmental, and illness factors on the ability to maintain sleep minute by minute across several nights of sleep in hospital. Results demonstrated that sound and light events are associated with increased hazard of waking in the night; further work is needed to decrease these environmental challenges to sleep for hospitalized children. Promising interventions that use parent-facilitated relaxation and comfort strategies to promote sleep and return to sleep from wakes^53,54^ or that optimize routine monitoring should be further explored.^55^ Our previous work^56,57^ has demonstrated sleep deprivation and effects on mood that may inhibit parents’ ability to support and care for their child during hospitalization. Development of future interventions should target the whole family and incorporate input from staff, administration, parents, and children.^58^ Strategies to decrease noise and light levels implemented with health care professionals are effective in the short term, but noise and light levels typically remain above recommended levels postintervention, indicating that a more comprehensive, multipronged approach is needed.^59^

### Strengths and Limitations

The present study took place at a single center, which limits generalizability to tertiary facilities in large, urban centers. Although this sample is one of the largest to date to use actigraphy to characterize sleep in hospitalized children, as well as light and noise in each child’s care environment, future work would be strengthened by larger samples and longer periods of sleep data collection. Most participants provided the minimum recommended 3 days and nights of sleep data recording; however, owing predominantly to early discharge, one-third of the sample had only 1 or 2 days and nights of recording. Although the present sample included children enrolled from both the PICU and general pediatrics units, we attempted to minimize differences introduced by these varied environments through statistical modeling that accounted for the location of sleep and environmental factors such as light, noise, and health care professional presence. In this sample, 87% of children had CSHQ scores indicative of problematic sleep at home, but as we found no association between CSHQ scores and minutes of sleep or risk of awakenings in hospital, this may reflect a need for further refinement of the tool, as others have suggested.^60^

Strengths of this study include a sample diverse in age, race, and reason for admission, with objective measures of sleep. We were able to synchronize observations of sleep, noise, and light, such that we were able to model associations of sleep duration and night wakings minute by minute. Future studies should add measures of room entries and care activities in the room,^61^ as well as tracking patient care interactions (eg, positioning, vital signs) and their effects on sleep.

## Conclusions

During hospitalization, children experience significant sleep restriction and frequent wakings from noise and light, almost certainly limiting their ability to cycle through all stages of sleep. Sound and light above recommended levels were associated with increased hazards of nighttime waking. When children do not achieve an adequate amount of sleep, they are at high risk for negative health outcomes, such as altered mood and cognitive deficits. Future interventions are needed that comprehensively decrease noise and light on pediatric hospital units.
